# Annotations

**Published:** 1905-03-04

**Authors:** 


### Annotations.

### Oysters and Bacilli.

All who have read the late Sir Henry Thomoson's book on "Food and Feeding," and, still more, all who were privileged to take part in the delightful "octave" dinners at which he was wont to give practical illustrations of the principles his writings were intended to enforce, will remember his admiration for the oyster, for the "half-dozen natives forming a single service of exquisite quality, with its attendant graces of mild and delicate vinegar or lemon-juice, brown bread and butter, and a glass of light chablis for those who take it." Not long before his death, and in relation, we think, to those mayoral banquets in the South of England which were followed by lamentable outbreaks of typhoid fever among the guests, Sir Henry expressed his conviction of the perfect safety of eating oysters which had been properly fed and treated after they were gathered. His opinions on this point seem to derive considerable support from some researches recently undertaken for the Worshipful Company of Fishmongers by Professor Klein, F.R.S., whose preliminary report, "On the Vitality of the Typhoid Bacillus in Shellfish," has just been published by the Company in pamphlet form. He placed clean Colchester oysters in sea water, in which typhoid bacilli were distributed to the amount of 744,000 bacilli per cubic centimetre. After 24 hours, oyster No. 1 was examined, and was found to contain in its body 40,000 bacilli. The other oysters were then placed in fresh clean sea water, and, after one day of this treatment, a second specimen yielded only 1,380 bacilli. After two days, a third specimen yielded 440; after five days another yielded 82; after four days another yielded 44; and, on subsequent days, no bacilli were discoverable. On the basis of this and several other experiments of like kind, Professor Klein formulates the conclusions that oysters readily take up the bacillus typhosus which has been introduced into the surrounding sea water, but that oysters rapidly clear themselves of the ingested bacilli if they are kept in clean sea water which is frequently changed. Oysters that have been long infected, or that are kept dry, do not clear themselves of bacilli with anything like the same rapidity; and there seems reason to suppose that the ultimate report, to be furnished when Professor Klein's researches have been concluded, will be likely to enable dealers so to treat their purchases as to secure consumers against risk.

### The Industrial Death-Roll.

At occasional intervals public attention, as the result of some appalling catastrophe, is directed to the risks which are encountered in the various industries that find occupation and livelihood for the artisan population of the country. In the absence of any such event, it is easy to forget how serious is the loss of life paid as an annual toll by members of the working-classes engaged in contributing an essential share in the organisation of the national life. The records of these personal and domestic disasters may be found in the Board of Trade returns, and they form in all truth a melancholy catalogue. It will doubtless come as a surprise to many that during the month of January, in the present year, the accidents officially reported involved no less than 402 deaths. Of these, 37 occurred in connection with railways, 103 in mines and quarries, 73 in workshops and factories, 26 in docks, wharves, etc., whilst 163 deaths were those of seamen. That these are not figures far in excess of the usual level is evident from the fact that the deaths chronicled under the same headings in January 1904 were 366, and in December of the same year 352—that is, in round numbers, a fatal list of something between 350 and 400 per month. Further, it must be remembered that these figures take no note of non-fatal injuries, which in many cases involve most serious consequences both to the worker and to those dependent on him. Another contribution to the same chapter is made by the cases of poisoning occurring in various industries. Among these in January 1905 were 49 instances of lead-poisoning, two of them attended with fatal results; in the corresponding month of the previous year the cases of lead-poisoning numbered 43, and there were also two cases of arsenical poisoning. These figures may well prompt reflection in many directions, and more particularly in regard to the question whether some proportion of these fatalities could not have been prevented. It is the custom to boast of the victories of peace, but, as is the case with the triumphs of war, there is need also to take note of the list of casualties.

### The Work of Special Hospitals.

The Hospitals Committee of the British Medical Association has formulated a number of recommendations on the general principles of hospital management, and among these is the proposition "that special hospitals shall treat only those cases that come strictly within the scope of their work." With this as a general statement there will, we think, be little disposition to quarrel. It is too much the practice at certain hospitals to accept almost every patient who presents himself so long as any one of his symptoms appears to come within the province which the hospital professes to cover. Hence cases of Graves' disease, for example, may be found in the clinics of hospitals devoted to diseases of the throat, or in those of ophthalmic hospitals. Or a case of bulbar paralysis may find its way to one of the former institutions, and an example of Bell's paralysis to one of the latter. Now, it may be allowed that, speaking generally, the proper course is to refer such patients to the care of a physician at a general hospital, as even though the members of a special staff are competent to deal with these diseases the hospital usually has not the appropriate equipment. Yet in practice the application of the rule presents many individual difficulties. Take as illustrations ocular paralyses, and defects of sight due to commencing optic atrophy or to the retinal changes which sometimes attend albuminuria. Patients in these groups find their way in large numbers to the special ophthalmic hospitals. Undoubtedly such conditions are outside the province of ophthalmic surgery, and demand the examination and care which can only be afforded by a member of the profession competent to deal with the general diseases of which the ocular facts are but an expression.

